# Is Surgery in Autoimmune Pancreatitis Always a Failure?

**DOI:** 10.3390/medicina59020193

**Published:** 2023-01-18

**Authors:** Hana Zavrtanik, Aleš Tomažič

**Affiliations:** Department of Abdominal Surgery, University Medical Centre Ljubljana, Zaloška Cesta 7, 1000 Ljubljana, Slovenia

**Keywords:** autoimmune pancreatitis, IgG4-related disease, pancreatic cancer, pancreatectomy, clinical decision-making

## Abstract

Autoimmune pancreatitis is a rare form of chronic pancreatitis of presumed autoimmune etiology. Due to significant overlap in clinical and imaging characteristics, misdiagnosis as a pancreatic malignancy is common. As a result, a significant number of patients undergo a major pancreatic resection, associated with considerable morbidity, for a disease process that generally responds well to corticosteroid therapy. In the past ten years, important advances have been made in understanding the disease. Several diagnostic criteria have been developed to aid in diagnosis. Despite this, pancreatic resection may still be required in a subset of patients to reliably exclude pancreatic malignancy and establish a definite diagnosis of autoimmune pancreatitis. This article aimed to define the role of surgery in autoimmune pancreatitis, if any. For this purpose, published case series of patients with a diagnosis of autoimmune pancreatitis, based on the histopathological examination of surgical specimens, were reviewed and patients’ clinical, radiological and serological details were assessed. At the end, histopathologic examinations of patients who underwent pancreatic resection at our department in the last 10 years were retrospectively reviewed in order to identify patients with autoimmune pancreatitis and assess their clinical characteristics.

## 1. Introduction

Autoimmune pancreatitis (AIP) was first described as a clinical entity in 1961 by Sarles et al. who described a type of sclerosing pancreatitis associated with hypergammaglobulinemia [1]. In 1995, Yoshida et al. summarized the clinical features of these patients and named the disease autoimmune pancreatitis [2]. Since then, its further clinical characterization has taken place, resulting in the development of the International Consensus Diagnostic Criteria (ICDC) for AIP [3]. According to these criteria, a diagnosis of AIP is based on five cardinal features; namely, pancreatic parenchymal and ductal imaging, serology, pancreatic histopathology, other organ involvement, and response to steroid therapy. Furthermore, two different histopathological patterns have been recognized with distinct clinical profiles [4]. The predominant type, histologically described as lymphoplasmacytic sclerosing pancreatitis, is a pancreatic manifestation of an IgG4-related systemic disease characterized by elevated serum IgG4 levels and the involvement of other organs due to abundant tissue infiltration of IgG4-positive plasma cells [5]. It shows a male predilection (3–4:1), peaking in prevalence in the 6th decade of life [6]. A final diagnosis can be made based on a combination of clinical features and frequently does not require histopathological confirmation [3]. In contrast, a different type of AIP not related to IgG4 and referred to as idiopathic duct-centric pancreatitis has been reported mainly in North America and Europe [7,8]. The primary histological feature of this type is the presence of a granulocytic epithelial lesion, characterized by focal disruption and destruction of the duct epithelium due to the invasion of neutrophilic granulocytes [7]. This type of the disease usually shows no or very few (<10 cells/HPF) IgG4-positive plasma cells and is not associated with serum IgG4 elevation or the involvement of other organs, as typically seen in lymphoplasmacytic sclerosing pancreatitis [6,9]. However, inflammatory bowel disease is commonly described in association with AIP type 2, with variable prevalence being reported among different studies and ranging from 10.4% to 100% [6,9,10,11,12]. The patients are on average a decade younger than patients with lymphoplasmacytic sclerosing pancreatitis and there is no sex predilection [6]. Because there is no serological biomarker or other organ involvement, a histological analysis of the pancreatic tissue is necessary for a definitive diagnosis [3,9]. Because histopathology is not always available, the terms type 1 and type 2 have been introduced to describe the clinical profiles associated with lymphoplasmacytic sclerosing pancreatitis and idiopathic duct-centric pancreatitis, respectively [3]. If the distinction between the two subtypes is not possible based on ICDC, these cases apply to the category of AIP-not otherwise specified (AIP-NOS). The diagnosis of AIP-NOS is therefore established as the standard diagnosis in the absence of conclusive criteria for the diagnosis of probable or definitive AIP type 1 as well as the absence of histological criteria for AIP type 2 or concurrent IBD [3]. Although the understanding of AIP has advanced significantly over the last decade, diagnosis of AIP remains challenging because the clinical and radiological presentation is often indistinguishable from pancreatic cancer [13]. As a result, a significant number of patients receive a major operative procedure for a disease process that generally responds well to corticosteroid therapy [14,15,16]. On the other hand, delay in diagnosis and treatment of pancreatic cancer has disastrous consequences, and prompt surgical treatment offers the only chance of survival [14]. These two diseases are most reliably distinguished by histopathological examination of resected specimens. Therefore, previously reported series of patients that underwent pancreatic resection for a presumed pancreatic malignancy but were diagnosed with AIP based on histology findings are of great research value for describing the histopathological, demographic, clinical, and radiological characteristics of AIP, as well as any diagnostic pitfalls.

This review discusses the potential role of surgery in the diagnosis and treatment of AIP, based on lessons learned from published surgical series. At the end, patients with AIP who underwent pancreatic resection at our department in the past 10 years were identified based on retrospective review of final histopathological examinations in order to assess their clinical characteristics.

## 2. Autoimmune Pancreatitis as a Diagnostic Challenge

Currently, the ICDC provides the most accurate guidelines for diagnosing AIP [3]. Despite well-defined diagnostic criteria, clinical and radiographic features that distinguish AIP from pancreatic cancer can be subtle and difficult to acknowledge. Patients with AIP typically present with obstructive jaundice, abdominal pain, and weight loss, which overlap considerably with the symptoms usually seen in pancreatic malignancy [17]. Furthermore, typical imaging findings of a diffusely enlarged pancreas, especially with a capsule-like rim, and irregular narrowing of the main pancreatic duct without upstream dilatation are frequently lacking. Instead, a focal inflammatory mass and gland atrophy, with or without ductal dilatation [13,17], is commonly revealed.

Serological biomarkers can potentially aid in definitive diagnosis and should be measured if IgG4-related gastrointestinal disease is suspected [18]. However, no specific biomarkers for AIP have been identified to date [19]. Serum IgG4 levels lack the sensitivity and specificity to establish a diagnosis of AIP or to distinguish it from other diseases such as primary sclerosing cholangitis, pancreatic cancer, and other hepatopancreatobiliary diseases [20,21]. Fold elevation above normal rather than absolute values is recommended, with an elevation >2 times the upper limit of normal being strongly suggestive of AIP in the setting of pancreatic mass [18,21]. Nevertheless, type 2 AIP is generally not associated with IgG4, and a proportion of type 1 AIP patients are seronegative [6,9]. Similarly, serum levels of CA 19-9, which is used as a tumor marker of pancreatobiliary malignancy, are influenced by other factors and can be elevated in benign biliary diseases presenting with jaundice and nonspecific causes of pancreatic inflammation. Therefore, the diagnostic accuracy of CA 19-9 in differentiating AIP from pancreatic cancer is limited when used alone [22,23].

The involvement of other organs as part of IgG4-related systemic disease is common in patients with type 1 AIP and has been demonstrated in the extrapancreatic bile ducts; salivary, lacrimal, and thyroid glands; kidneys; lungs; mediastinal and abdominal lymph nodes; the aorta; and the retroperitoneum [24]. Patients may therefore present with obstructive jaundice due to IgG4-related sclerosing cholangitis causing bile duct strictures, bilateral lacrimal and salivary gland swelling due to dacryo- and sialadenitis, or lymphadenopathy, either generalized or localized. Alternatively, they may develop tubulointerstitial nephritis, pneumonitis, aortic aneurysms or dissections due to aortitis, mediastinal or retroperitoneal fibrosis [24]. Extrapancreatic manifestations can be diagnosed by imaging, clinical examination, or histological evaluation of the affected tissue. The well-described pattern of other organ involvement is an important clue and suggests AIP rather than pancreatic cancer [3]. When present, it strengthens the diagnosis of AIP and possibly allows for histological confirmation, especially since some other tissues are readily amenable to biopsy [3]. However, extrapancreatic manifestations do not necessarily occur simultaneously but may precede or be subsequent to AIP, therefore not pointing toward a diagnosis of AIP at the time [24]. Again, type 2 AIP has no relation to IgG4 and usually lacks the involvement of other organs, but it should be strongly considered in the presence of concurrent inflammatory bowel disease [9,10].

Finally, the two types of AIP display a typical pathohistological pattern; however, histology is not usually available because adequate pancreatic tissue is difficult to obtain [3]. Tissue obtained by endoscopic ultrasound (EUS) fine-needle aspiration (FNA) may not be representative, given the limited caliber of the needle and architectural distortion [25,26]. Moreover, the results are commonly suggestive of pancreatic cancer demonstrating ductal atypia [27,28]. Therefore, EUS FNA is not recommended for diagnosing AIP according to ICDC guidelines. Instead, tissue acquisition from either surgical resection or a pancreatic core biopsy specimen may be used [3,29]. Core biopsies, conducted through EUS-trucut biopsy (EUS-TCB) or newer-generation EUS fine-needle biopsy (EUS-FNB), enable greater tissue specimen size and better histopathologic review [29]. According to European guidelines on IgG4-related digestive disease, EUS may demonstrate hypoechoic pancreatic enlargement and other features suggestive of AIP. Furthermore, it is used for obtaining tissue samples for histological diagnosis [30]. EUS-guided tissue acquisition with a core biopsy using a 19-gauge needle is recommended; however, even a 22-gauge needle can be used to obtain a sample allowing for histological evaluation [30,31]. Additionally, IgG4 immunostaining of biopsy specimens from the major papilla may advance a diagnosis of AIP as it is often involved [3,30,32]. Future advances in endoscopic technologies, enabling them to take sufficiently large biopsy samples, will likely improve the diagnostic process. New types of EUS-guided needles for adequate pathological samples have now been developed, but their diagnostic utility is yet to be evaluated in future prospective studies [29].

In patients with a suspicion of AIP and negative work-up for cancer including EUS FNA, a diagnostic steroid trial may be helpful in confirming the diagnosis of AIP [3]. Administration of high-dose prednisone (0.6–1 mg/kg/day) is followed by a reassessment of imaging and CA 19-9 after 2–4 weeks of treatment. Rapid definite improvements in imaging abnormalities and a decrease in CA 19-9 levels, if elevated before treatment, are expected [24,30,33]. After treatment response, the prednisone dose is tapered by 5 mg each week until discontinuation [24,30]. In case of poor response to steroid therapy, the diagnosis should be reconsidered. However, a steroid trial needs to be conducted with caution in well selected patients since various conditions, including peritumoral pancreatitis, potentially respond to immunosuppression [3].

## 3. Surgical Experience in Autoimmune Pancreatitis

Given the diagnostic challenges described above, pancreatic resection may be required to exclude pancreatic malignancy and establish a diagnosis of AIP. Among pancreatic resections performed due to suspected malignancy, benign conditions have been found in 8 to 10% of patients upon final histological examination. Among these, AIP represents about a third of cases, accounting for 2.5% of all pancreatic resections [15,16,34,35]. These cases should be prevented, since the postoperative course after pancreatic resections can be marked by several life-threatening complications. The most significant one is pancreatic fistula, with its septic and hemorrhagic sequelae. Various modalities, such as several modifications of anastomotic techniques, the placement of pancreatic duct stents and prophylactic use of somatostatin analogues, have been implemented to improve postoperative outcomes. Although perioperative mortality has decreased significantly with gaining surgical experience and critical care management, morbidity rates remain high, even in high-volume centers [36,37]. Therefore, adequate preoperative workup is crucial in order to improve patient selection to avoid unnecessary surgery. A review of surgical case series published in the past 10 years clearly demonstrates the difficulties in AIP diagnosis (Table 1). Patients with AIP that were candidates for surgery frequently lacked the typical findings of AIP. Therefore, AIP was not necessarily suspected before surgery, which resulted in an insufficient preoperative workup. For example, only a few studies reported preoperative serum IgG4 levels, which were measured in a range from 14 to 57% of the study cohort [15,34,35,38,39,40]. Ikeura et al. determined serum IgG4 levels to be present in 77% of the study cohort; however, these are cumulative measurements, taken before or after surgery [41]. Furthermore, serum levels of IgG4 were generally not helpful in differential diagnosis between AIP and malignant lesions because they were elevated in only about a third of patients in whom AIP type 1 was pathologically confirmed after surgery [15,34,38,40]. In contrast, CA 19-9 was more commonly assessed, and elevated CA 19-9 levels were found in 25 to 52% of patients [15,34,40,42]. Javed et al. even reported a greater proportion of patients with AIP that presented with elevated CA 19-9 than with elevated IgG4 (47.1 vs. 26.1% for CA 19-9 and IgG4, respectively) [34]. Preoperative tissue sampling was performed in 0 to 73% of patients, among whom suspicion of malignancy was reported in 5 to 25%, although they eventually proven to be false positives [15,34,35,39]. However, histopathological confirmation of a malignant disease is not necessary before proceeding with surgical resection of a pancreatic solid mass according to the International Study Group of Pancreatic Surgery [14]. Again, limited data on preoperative tissue sampling may indicate the lack of preoperative suspicion of AIP. The presence of extrapancreatic manifestations of IgG4-related systemic disease may to help raise the suspicion of AIP; however, these were reported in 13 to 71.4% of cases and sometimes occurred only after surgery had already been performed [15,35,40,41,43].

Interestingly, despite expanding knowledge, the number of patients with AIP undergoing pancreatic resection and therefore being misdiagnosed with pancreatic malignancy did not decline over time in the studies reported [15,39,40].

Further highlighting the challenges in diagnosing AIP, Ikeura et al. found the diagnostic yield of ICDC without histology and response to steroids to be quite low in focal AIP patients suspected of having cancer, diagnosing AIP in only 20% of patients. A steroid trial after ruling out pancreatic cancer improved the diagnostic yield of ICDC to 73% despite the lack of histology, indicating the important role of pancreatic core needle biopsy or surgical resection in the remaining patients [41].

Importantly, a high index of clinical suspicion for AIP is needed to improve patient selection for surgery. To avoid unnecessary surgery or diagnostic procedures, it is crucial to consider the possibility of AIP at an early stage of patient workup. Van Heerde et al. found that a routine workup for pancreatic cancer is not enough to detect these patients, and they suggested considering IgG4 measurement and the systematic use of diagnostic criteria systems in every patient eligible for pancreatic resection without preoperative histological confirmation of malignancy [15]. When AIP is highly suspected, measurement of serum levels of IgG4 and a biopsy are recommended, with EUS-guided trucut biopsy being a preferred modality. If the biopsy is not diagnostic or suspicious for malignancy, a short 2-week course of steroid treatment could be an alternative to surgery. Rapid (≤2 weeks) resolution in imaging abnormalities is seen in cases of AIP. On the other hand, in cases of an eventual malignant lesion, no change in operability is expected during this short period [3]. However, even with aggressive preoperative evaluation, there is still a small subset of patients in whom malignancy cannot be excluded without pancreatic resection, which remains necessary to provide a definite diagnosis of AIP.

## 4. Consideration of Surgical Treatment in Autoimmune Pancreatitis

The mainstay of AIP treatment is corticosteroid therapy, which induces remission in more than 90% of patients with AIP [8,33,44,45]. Nonetheless, relapses are common, and there is no clear consensus regarding treatment in relapsed cases [8,46,47].

The role of surgical resection in the management of AIP is controversial. As mentioned above, it is most certainly indicated in cases when pancreatic malignancy cannot be excluded after a complete diagnostic workup [18]. Apart from that, it can potentially result in good symptom control and the resolution of associated biliary strictures [47]. Some authors therefore suggest that surgery in AIP may be indicated in patients with poor responses to steroid therapy in whom long-term biliary stenting would be necessary due to continuous obstructive jaundice or in the presence of highly symptomatic repeated relapses [39,42,47]. In a study by Schnelldorfer et al. long-term pain control and improvement in quality of life appeared to be good with operative intervention alone, stressing the validity of operative intervention for AIP in selected cases [48]. Miura et al. and Dickerson et al. found no relapse of AIP after pancreatic resection [38,42]. Whereas other authors reported relapse rates ranging from 10.7 to 57% for type 1 AIP, the postoperative relapse of AIP was much less common in patients with type 2 AIP (0–15.4%) [34,39,40,41,43]. Similarly, a few other studies demonstrated that patients after palliative pancreatic resection or bypass can achieve successful clinical remission and rarely relapse, especially in type 2 AIP [6,8,33,38,42]. In a study by Sah et al., 47% of patients with type 1 and none with type 2 experienced a relapse, whereas pancreatic resection significantly reduced the risk of relapse in patients with type 1 AIP [6]. However, when the diagnosis of AIP is certain and malignancy is excluded, treatment with rituximab could be a more efficient alternative and resolve the biliary manifestations without surgery [46]. Adding confusion to clinical decision making, some AIP cases were found with concurrent pancreatic cancer and cancer precursor lesions in resected specimens [34,35,40,42]. Macinga et al. reported a surprisingly high rate (40%) of pancreatic cancer in patients with AIP that underwent pancreatic resection due to focal pancreatic mass [35]. In series by Javed et al. synchronous AIP and infiltrating pancreatic ductal adenocarcinoma were found upon resection in one patient [34,49]. Furthermore, incidental pancreatic intraepithelial neoplasias (PanINs) were found in nearly a quarter of patients included in their study [34]. An even greater prevalence (82%) of PanIN lesions among pancreatic resection specimens with a final histopathologic diagnosis of AIP, was demonstrated in a study by Gupta et al. [50]. As precursor lesions of invasive ductal carcinoma, PanINs likely represent an elevated risk of developing pancreatic cancer [51]. These findings suggest that surgical resection is reasonable in this subset of patients with AIP. Similarly, cases of a synchronous diagnosis of AIP and pancreatic cancer have been repeatedly described in the literature [20,45,52,53,54]. A recent scoping review, regarding the incidence of pancreatic cancer in patients with AIP, identified 11 patients with synchronous occurrence of AIP and pancreatic cancer [35]. Although the relationship between the two diseases is not completely understood, clinicians should be aware that a preoperative diagnosis of AIP does not rule out the simultaneous presence of pancreatic cancer. A possible diagnosis of pancreatic cancer needs to be thoroughly excluded in patients in whom AIP is suggested based on clinical and image findings, or even when a diagnosis of AIP is confirmed [35,49,53]. Possible synchronous occurrence of AIP and pancreatic cancer entails major clinical consequences, requiring greater caution when opting to manage AIP patients without surgical resection.

## 5. Our Experience

Histopathologic examinations of pancreatic resection specimens from patients who underwent surgery at our department during 2012–2022 were retrospectively reviewed. Electronic patient records of three patients who were found to have AIP upon final histopathologic examination were evaluated to further determine their clinical characteristics.

The first patient was a 73-year-old female who presented with obstructive jaundice. A 4 × 4 cm large tumor in the head of the pancreas with uneven enhancement, infiltrating the duodenal wall, was found upon the performance of an abdominal CT scan. Levels of tumor markers CA 19-9 and CEA were normal, while IgG4 levels were not assessed. The patient underwent pancreatoduodenectomy with an uneventful postoperative course. Histopathologic examination revealed histopathologic patterns typical of AIP type 1, with dense periductal lymphoplasmacytic infiltration, storiform fibrosis, focal obliterative arteritis and abundant (78 per HPF) infiltration of IgG4 plasma cells. The patient was referred to a gastroenterologist and was regularly followed up with afterwards, showing noclinical signs of relapse.

The second patient was a 46-year-old male who presented with mild unspecific abdominal pain. Laboratory values showed hyperbilirubinemia, with elevated levels of transaminases and alkaline phosphatase. Besides, he was newly diagnosed with diabetes. Abdominal US revealed a 4 cm hyperechoic mass in the head of the pancreas extending towards its neck and body. Upon abdominal CT scan, a diffusely enlarged pancreas of homogenous structure was seen, with no dilatation of pancreatic or biliary ducts. However, the narrowing of the distal choledochal duct together with structural changes to the pancreatic body and tail were described upon EUS and the specimen obtained by EUS FNA from the pancreatic head was interpreted as poorly differentiated adenocarcinoma with signet ring cell morphology. Tumor marker CA 19-9 levels were mildly elevated (77 kU/L, normal value up to 37 kU/L), together with slightly elevated levels of CEA and IgG4. After ERCP with brush cytology, which was unrepresentative, and stent placement, this patient underwent pancreatoduodenectomy. During the surgery, he suffered from massive hemorrhage from superior mesenteric–portal vein confluence, due to which he was admitted to intensive care unit for a day. The rest of the postoperative course was uneventful. Final histopathologic examination showed a histopathologic pattern typical of AIP type 1 with periductal lymphoplasmacytic infiltration, storiform fibrosis and obliterative phlebitis. The patient was referred to a gastroenterologist at a peripheral hospital and was lost to follow-up.

The third patient was a 52-year-old female who was found incidentally to have two cystic pancreatic lesions upon abdominal US. She therefore underwent MRCP that showed tortuous dilatation of main pancreatic duct up to 8 mm in the pancreatic head, up to 9 mm in the pancreatic neck and up to 6 mm in the pancreatic body, suggestive of main duct IPMN together with 12 mm branch duct IPMN in the uncinate process. Tumor markers CA 19-9 and CEA were normal, whereas IgG4 levels were not assessed. Upon a multidisciplinary team meeting, surgical resection was proposed. Pancreatoduodenectomy was performed with uneventful recovery, excepting for the diabetes that she developed after the procedure. Histopathologic examination showed histopathologic pattern typical of AIP type 2 with extensive storiform fibrosis and granulocyte epithelial lesions. Mild cystic dilatation of pancreatic ducts was also observed with no signs of tumor cells. The patient is still followed at the outpatient clinic, with no clinical signs of relapse.

An evaluation of the described cases further highlights the challenges clinicians face in AIP diagnosis and management. For example, the second patient was found with typical imaging findings of AIP. However, EUS FNA pointed towards a completely different diagnosis, for which he underwent surgical resection and suffered from life-threatening intraoperative complications. In our series, a low index of suspicion of AIP due to unspecific clinical presentation and various imaging characteristics was a main problem in recognizing the disease, resulting in an inadequate preoperative workup, lacking for example a thorough assessment of other organ involvement and IgG4 levels.

## 6. Conclusions

Autoimmune pancreatitis is a rare pancreatic disorder that requires a multidisciplinary approach to diagnosis and management. A greater level of awareness and knowledge regarding clinical manifestations of AIP, as well as the routine use of ICDC, would allow for earlier diagnosis of the disease and help avoid unnecessary diagnostic procedures and major pancreatic surgery for unrecognized AIP. Primary treatment for AIP should be nonoperative. However, we believe surgical resection may be necessary in a subset of AIP patients with (1) unclear diagnosis and inability to exclude pancreatic malignancy after a complete diagnostic workup, (2) a poor response to steroid therapy or highly symptomatic repeated relapses, and (3) the coincidence of AIP and pancreatic cancer.

## Figures and Tables

**Table 1:** Review of studies published in the last decade regarding patients with autoimmune pancreatitis after pancreatic resection.

Study (Study Period)	Study Cohort	AIP Type	Symptoms	Imaging	IgG4 ^a^	CA 19-9 ^a^	Preoperative Biopsy ^a^	Concurrent Malignancy	IgG4-Related OOI	Follow-Up	Relapse
Detlefsen, 2012 (1987–2010)	114 pts with AIP	1: 63 (55.3%) 2: 51 (44.7%)	NS	NS	Assessed in 29 pts (25.4%) Postop ↑ in 1: 12/18 (66.7%) 2: 1/11 (9.1%)	NS	NS	None	1: 33/63 (52.4%) 2: 6/51 (11.8%)	5.3 y (mean)	1: 21/51 (41.2%) 2: 6/39 (15.4%)
van Heerde, 2012 (2000–2009)	7 pts with AIP among 274 PDs (2.6%)	1: 2 (28.5%) 2: 4 (57.2%) AIC: 1 (14.3%)	Jaundice 86% Weight loss 2.7 kg (mean) Abdominal pain 29% Recent onset diabetes (29%)	No typical imaging findings were revealed in any of the patients	Preop ↑ in 1/3 (33%/42.9%)	Preop ↑ in 3/7 (43%/100%)	EUS FNA performed in 1 pt which was suspicious for malignancy (14.3%)	NS	1: 2 (100%) 2: none	NS	NS
Miura, 2013 (1990–2010)	13 pts with AIP compared to 29 pts with CP	All type 1	Abdominal pain (46%) Jaundice (23%)	Focal MPD stricture 10 pts (77%) MPD dilatation 6 pts (46%) Pancreatic enlargement 8 pts (62%, 5 focal, 3 diffuse)	Preop ↑ in 1/3 (33%/23.1%) Postop ↑ in 2/3 (66%/23.1%)	NS	NS	None	None	45.6 mo (median)	None
Clark, 2013 (1986–2011)	74 pts with AIP	1: 34 (46%) 2: 29 (39%) Unknown: 11 (15%)	Jaundice (46%) Weight loss (45%) Pancreatitis (34%)	MPD dilatation 24 pts (32%) Pancreatic enlargement-diffuse 14 pts (19%) Pancreatic mass 52 pts (70%)	Preop assessed in 10 (14%), 64 mg/dL (median)	Preop assessed in 41 (55%), 17 U/mL (median)	4 pts (5%) had preoperative biopsy indicating malignancy	None	NS	58.4 mo (median)	Total: 11/66 (17%) 1: 7 (25%) 2: 3 (11%)
Ikeura, 2014 (1996–2012)	30 pts with AIP	1: 23 (77%) 2: 7 (23%)	Jaundice (57%) Abdominal pain (37%) Weight loss (33%)	Long or multiple MPD strictures 9 pts (30%) Focal MPD stricture 13 pts (43%) Pancreatic enlargement-focal 19 pts (63%) Pancreatic mass 11 pts (37%) Capsule-like rim 1 pt (3%)	Pre- or postop ↑ in 11/23 (48%/76.7%) 3 pts (13%) >2x upper limit	NS	None	None	1: 8 (35%)—3 pts developed OOI after surgery	NS	1: 4 (57%) 2: none
Vitali, 2014 (2005–2011)	11 pts with AIP among 373 pancreatic resections (2.9%)	1: 8 (73%) 2: 3 (27%)	Abdominal pain (64%) Weight loss (64%) Jaundice (36%) Acute pancreatitis (27%)	NS	NS	NS	NS	None	NS	NS	NS
Macinga, 2017 (2000–2013)	15 pts with AIP among 295 pancreatic resections (5.1%)	1: 6 (40%) 2: 9 (60%)	Weight loss (80%) Jaundice (47%) Recent onset diabetes (33%)	Atypical imaging findings 9 pts (60%) L2 evidence acc. to ICDC 6 pts (40%)	Preop normal in 1: 3/3 (100%/20%)	Pts without PDAC 35.2 kU/L (mean) Pts with PDAC 89.8 kU/L (mean)	EUS FNA performed in 11 pts (73.3%) True positive 3 pts (27.3%, AIP + PDAC) Inconclusive 2 pts (18.2%, 1 with AIP + PDAC) True negative 4 pts (36.4%) False positive 2 pts (18.2%)	6 (40%) with PDAC	1: 2 (13%)—after surgery	NS	NS
Dickerson, 2019 (1997–2016)	45 pts with AIP, 27 underwent surgery	1: 16 (59.3%) 2: 9 (33.3%) NOS: 2 (7.4%)	NS	MPD stricture 5 pts (20%) MPD dilatation 3 pts (12%) Pancreatic enlargement-focal 6 pts (24%), diffuse 14 pts (56%) Pancreatic mass 4 pts (16%) Capsule-like rim 0 pts (0%) Double duct sign 3 pts (12%)	↑ in 2/11 (18.2%/24.4%)	↑ in 12/26 (46.2%/96.3%)	Of the EUS FNA performed, none confirmed or suggested AIP	3 pts (11.1%)—2 with PDAC and 1 with solid pseudopapillary neoplasm	NS	NS	None
Javed, 2021 (2001–2006)	56 pts with AIP among 5709 pancreatic resections (0.9%) compared to 32 pts with AIP managed conservatively	All type 1	Jaundice (64.3%) Abdominal pain (62.5%) Weight loss (53.6%)	MPD dilatation 15 pts (26.8%) Pancreatic enlargement 16 pts (28.6%) Pancreatic mass 32 pts (57.1%) Pancreatic atrophy 6 pts (10.7%)	↑ in 6/23 (26.1%/41.1%), 34.1 mg/dl (median)	↑ in 16/31 (51.6%/55.4%), 40 U/mL (median)	Preoperative biopsy in 38 pts (67.9%) Nondiagnostic in 18 pts (51.4%) PDAC in 11 pts (31.4%) CP in 4 pts (11.4%) Cellular atypia in 3 pts (8.6%)	1 pt with occult PDAC, 15 pts (27.3%) with PanINs	NS	14.8 mo (median)	6 (10.7%)
Nikolic, 2022 (2001–2020)	159 pts with AIP, 35 underwent surgery	1: 28 (80%) 2: 7 (20%)	Abdominal pain (48.6%) Weight loss (48.6%) Jaundice (42.9%)	MPD stricture 1 pt (2.9%) Pancreatic mass 19 pts (54.3%) Double duct sign 1 pt (2.9%)	Preop ↑ in 7/20 (35%/20%) 1 pt (5%) >3x upper limit	Preop ↑ in 5/20 (25%/57.1%)	EUS FNA performed in 2 pts—1 with benign features, 1 inconclusive Brush cytology performed in 10 pts with benign features (28.6%)	8 pts (22.9%)—2 with PDAC, 2 with MCN (1 with HGD), 4 with IPMN (1 with HGD)	25 (71.4%)	50 mo (median)	1: 9 (36%) 2: none

1: type 1. 2: type 2. AIC: autoimmune cholangitis. AIP: autoimmune pancreatitis. CP: chronic pancreatitis. EUS FNA: endoscopic ultrasound-guided fine-needle aspiration. HGD: high-grade dysplasia. ICDC: International Consensus Diagnostic Criteria. IPMN: intraductal papillary mucinous neoplasm. MCN: mucinous cystic neoplasm. MPD: main pancreatic duct. NOS: not otherwise specified. NS: not specified. PanIN: pancreatic intraepithelial neoplasia. PDAC: pancreatic ductal adenocarcinoma. PD: pancreatoduodenectomy. ^a^ percentage of elevated and assessed cases are calculated.

## Data Availability

Not applicable.
